# Valve-in-valve transcatheter aortic valve implantation (TAVI): a new valuable approach to bioprosthetic infective endocarditis?

**DOI:** 10.1093/ehjcr/ytae384

**Published:** 2024-07-30

**Authors:** Joelle Kefer, David Vancraeynest, Véronique Roelants, Leila Belkhir

**Affiliations:** Division of Cardiology, Cliniques universitaires Saint-Luc, Université Catholique de Louvain (UCLouvain), Avenue Hippocrate, 10-2881, 1200 Brussels, Belgium; Pôle de Recherche Cardiovasculaire, Institut de Recherche Expérimentale et Clinique (IREC), Université Catholique de Louvain (UCLouvain), Avenue Hippocrate, 10-2881, 1200 Brussels, Belgium; Division of Cardiology, Cliniques universitaires Saint-Luc, Université Catholique de Louvain (UCLouvain), Avenue Hippocrate, 10-2881, 1200 Brussels, Belgium; Pôle de Recherche Cardiovasculaire, Institut de Recherche Expérimentale et Clinique (IREC), Université Catholique de Louvain (UCLouvain), Avenue Hippocrate, 10-2881, 1200 Brussels, Belgium; Department of Nuclear Medicine, Cliniques universitaires Saint-Luc, Université Catholique de Louvain (UCLouvain), Brussels, Belgium; Department of Infectious Disease, Cliniques universitaires Saint-Luc, Université Catholique de Louvain (UCLouvain), Brussels, Belgium

A 77-year-old female patient was admitted for heart failure and severe aortic Magna Ease 21 mm bioprosthetic valve dysfunction (*Figure 1A*, Supplementary material online, *Movie Image S1*). Echocardiography revealed a vegetation on the right cusp of the bioprosthesis (*Figure 1B*, Supplementary material online, *Movie Images S2* and *S3*), and blood cultures were positive for a *Streptococcus sanguinis*. Despite antibiotics (ceftriaxone followed by penicillin), stroke due to septic embolization occurred 3 days after the initial presentation.

**Figure 1:**
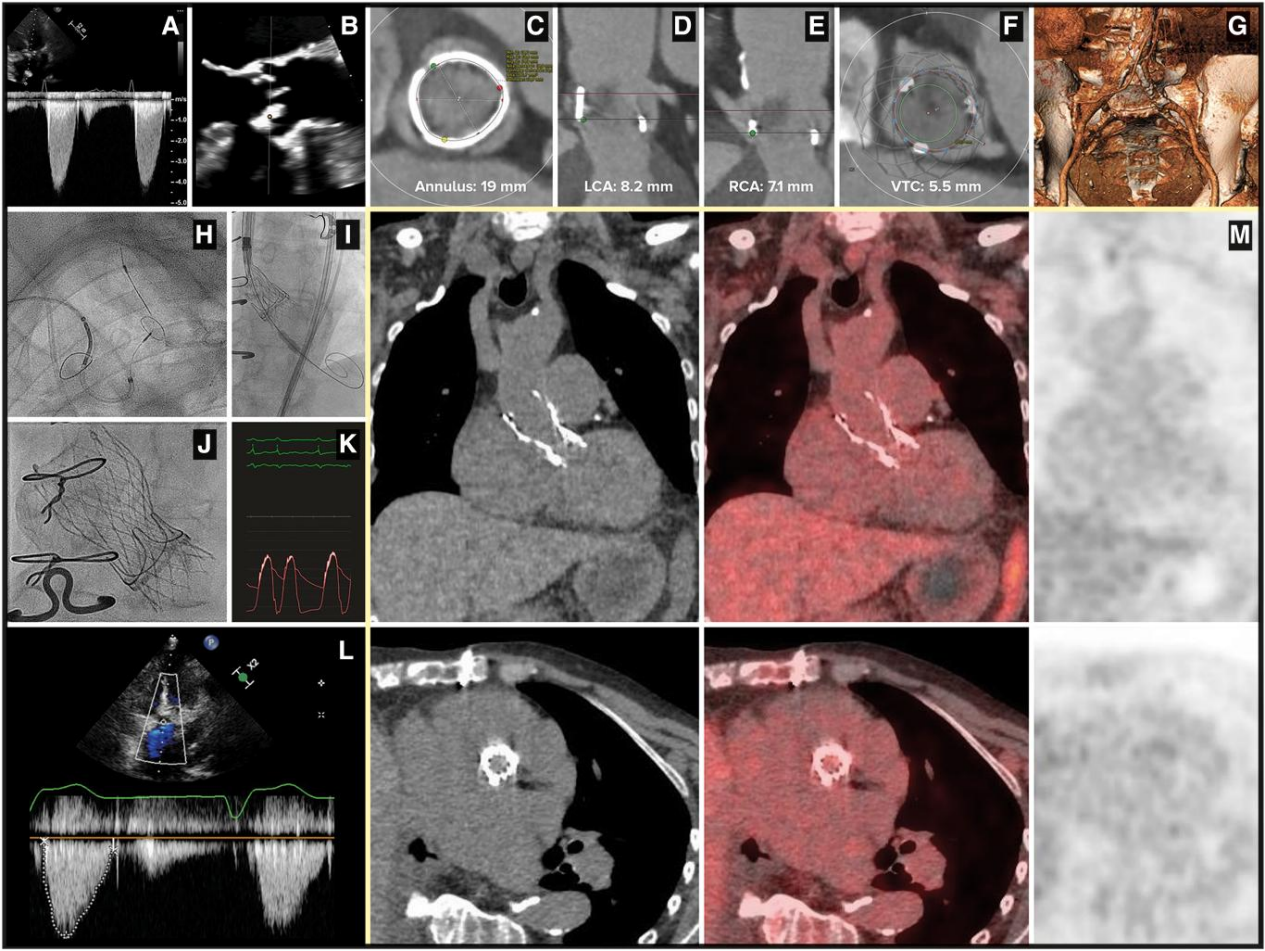
(*A*) Transvalvular aortic peak gradient obtained by transthoracic echocardiography. (*B*) Vegetation on the right cusp of the bioprosthetic valve at transoesophageal echocardiography. (*C*) Aortic annulus dimensions by CT-scan. (*D*) Distance between annulus and left coronary artery (LCA) ostium measured by CT-scan at 8.2 mm. (*E*) Distance between annulus and right coronary artery (RCA) ostium measured by CT-scan at 7.1 mm. (*F*) Virtual distance between transcatheter valve and coronary ostium (VTC) measured by CT-scan at 5.5 mm. (*G*) Femoral vascular access by CT-scan. (*H*) A cerebral protection device Sentinel deployed by the right radial access. (*I*) Fluoroscopic aspect of the Evolut-R 23 mm deployed in a Magna Ease 21 mm using the valve-in-valve technique. (*J*) Fluoroscopic aspect of the Evolut-R 23 mm implanted in the Magna Ease 21 mm. (*K*) Haemodynamic invasive transvalvular gradient after TAVI. (*L*) Transvalvular aortic gradient at follow-up after TAVI assessed by transthoracic echocardiography. (*M*) Absence of abnormal F-18-fluorodeoxyglucose uptake at PET-CT at follow-up after TAVI.

The recommended treatment^1^ would have been surgical intervention, for a complete debridement of the infected material. Because of a prohibitive risk for surgery, the heart team considered a transcatheter aortic valve implantation (TAVI), which has not been empirically considered in this context until now, mainly due to the procedural risk and to the expected probability of infectious relapse during follow-up.

According to the pre-procedural computed tomography (CT) findings (*Figure 1C–G*), an Evolut-R 23 mm was successfully implanted (*Figure 1H–J*, and Supplementary material online, *Movie Image S4*). Thanks to the supra-annular design of the Evolut platform, the peak residual gradient was only 4 mmHg, with no leak (*Figure 1K*, Supplementary material online, *Movie Image S5*).

The periprocedural period was uneventful, antibiotics were stopped at Day 3, and patient was discharged at Day 8 under apixaban, with a good clinical outcome after 3 months, a well-functioning valve (peak gradient of 17 mmHg, trivial leak, no vegetation, no abscess—*Figure 1L*). There was no reinfection, as suggested by the low level of C-reactive protein (0.8 mg/dL—normal ranges: 0.6–1.3) and the absence of abnormal uptake during 18F-FDG PET-CT (*Figure 1M*).

To date, only six male patients underwent TAVI for active aortic valve infective endocarditis.^2^ To our knowledge, this is the first report of TAVI performed in a frail woman to treat a bioprosthetic infective endocarditis, with a PET-CT follow-up illustrating the absence of infectious relapse. Since surgical aortic valve replacement is not offered to a substantial number of patients with prohibitive surgical risk, TAVI would become a valuable approach to correct the residual valvular dysfunction despite antibiotics. The heart teams could include this strategy in the decisional tree of bioprosthetic infective endocarditis, integrating it in the lifetime management^3^ of each patient.

## Supplementary Material

ytae384_Supplementary_Data

## Data Availability

Authors confirm that they make available the underlying deidentified data on which their research relies to the Journal for inspection and verification during the peer review process.

## Supplementary material

**Consent**: The patient signed the consent form for publication of the case report, according to the COPE guidelines and we confirm that we have this written consent.

**Funding**: This research did not receive any specific grant from funding agencies in the public, commercial, or not-for-profit sectors.
